# Effect of Antioxidant Mixtures on Growth and Ochratoxin A Production of *Aspergillus* Section *Nigri* Species under Different Water Activity Conditions on Peanut Meal Extract Agar

**DOI:** 10.3390/toxins2061399

**Published:** 2010-06-10

**Authors:** Carla Barberis, Andrea Astoreca, María Guillermina Fernandez-Juri, Ana María Dalcero, Carina Magnoli

**Affiliations:** Departamento de Microbiología e Inmunología, Facultad de Ciencias Exactas, Físico-Químicas y Naturales, Universidad Nacional de Río Cuarto, Ruta Nacional Nº 36 Km 601 (5800) Río Cuarto, Córdoba, Argentina; Email: cbarberis@exa.unrc.edu.ar (C.B.); aastoreca@exa.unrc.edu.ar (A.A.); jfernandezj@exa.unrc.edu.ar (M.G.F.-J.); adalcero@exa.unrc.edu.ar (A.M.D.).

**Keywords:** butylated hydroxyanisol, propyl paraben, growth parameters, ochratoxin A, *Aspergillus carbonarius*, *A. niger* aggregate, peanut meal extract agar

## Abstract

The effect of mixtures of antioxidants butylated hydroxyanisol (BHA) and propyl paraben (PP) on lag phase, growth rate and ochratoxin A (OTA) production by four *Aspergillus* section *Nigri* strains was evaluated on peanut meal extract agar (PMEA) under different water activities (a_w_). The antioxidant mixtures used were: BHA + PP (mM), M1 (0.5 + 0.5), M2 (1.0 + 0.5), M3 (2.5 + 0.5), M4 (0.5 + 1.0), M5 (1.0 + 1.0), M6 (2.5 + 1.0), M7 (5.0 + 2.5) and M8 (10 + 2.5). The mixture M8 completely suppressed mycelial growth for all strains. A significant stimulation in OTA production was observed with mixtures M1 to M5 mainly at the highest a_w_; whereas M6, M7 and M8 completely inhibited OTA production in all strains assayed; except M6 in *A. carbonarius* strain (RCP G). These results could enable a future intervention strategy to minimize OTA contamination.

## 1. Introduction

Peanut (*Arachis hypogaea* L.) is an important oilseed crop, and a major food legume, cultivated in over 100 tropical and subtropical countries. The seed has several purposes as whole seed or processed to make peanut butter, oil, soups, stews and other products. The cake has several uses in feed and infant food formulations. The protein, oil, fatty acid, carbohydrate and mineral content of this nut become sensitive to fungal contamination, in pre and post-harvest stage. The fungal contamination is one of the main problems when inappropriate processing and storage conditions occur [1].

This oilseed is one of the most important agricultural products in the Argentinean economy. The center-south region of Córdoba province produces 94% of the country’s production. The peanut industry exports 90% of its product, with Argentina being the second in the world in peanut exports. This activity is not a production chain, but meets all characteristics of a cluster such as geographical proximity, expertise and innovation [2]. At the post-harvest stage around 8% loss of the total production by peanut disease and mycotoxins contamination has been reported in recent years [3].

*Aspergillus* species are important contaminants of several pre, post harvest and stored cereal and oilseed grains. Furthermore toxigenic species of *Aspergillus* section *Flavi* and *Nigri* and the main mycotoxins (aflatoxins and ochratoxin A) have been detected in different nuts e.g., peanut kernels [4,5,6,7]. *Aspergillus* section *Nigri* species have acquired interest by their ability to produce ochratoxin A (OTA), a potent nephrotoxin known for the teratogenic, immunosuppressive and carcinogenic effects. It has been classified by the International Agency for Research on Cancer [8] as a possible human carcinogen (group 2B) based on sufficient evidence of carcinogenicity for animals and inadequate evidence in humans [9].

In our region, the presence of potential OTA-producer species has been recently detected in wine grapes, dried vine grapes, corn and stored peanut kernels [6,7,10,11,12].

Synthetic antioxidants, namely, food grade antioxidants and antimicrobials [13], are products widely used as preservatives especially in foods that contain oils or fats because they exhibit an exceptional stress oxidative protection. At present, butylated hydroxyanisole (BHA) and propyl paraben (PP) are permitted for use as antimicrobial agents in different foods and are on the list generally regarded as safe (GRAS) chemicals of the Food and Drug Administration in the USA. Several phenolic antioxidants showed *in vitro* biocidal action against yeast [14] and filamentous fungi [15]. These compounds showed the capacity to control mycotoxigenic fungi growth and mycotoxin accumulation in synthetic media and agricultural products such as corn and peanut kernels [16,17,18,19,20,21,22,23,24,25,26,27].

In previous studies, the effectiveness of BHA, butylated hydroxytoluene (BHT), and PP as fungal inhibitors in relation to *A. flavus* and *A. parasiticus* strains and their toxin accumulation on peanut meal extract agar has been determined. In these studies a fungal control was observed when these antioxidants and antimicrobial were applied in binary mixtures [23,24]. On the other hand, Reynoso *et al.* [17] observed that the binary mixtures of BHA and PP were effective to reduce the growth rate and fumonisin production by *Fusarium verticillioides* and *F. proliferatum* in corn meal extract agar. 

Recently, the effect of BHA and PP alone over a wide range of concentrations (1 to 20 mM) on the growth rate and OTA production by the *Aspergillus* section *Nigri* species on peanut meal extract agar at three water activities was evaluated [28,29]. The results of those studies suggest that growth rate and OTA production by these strains are completely inhibited at concentrations of 20 and 5 mM of BHA and PP, respectively. However, there is no available information on the efficacy of antioxidants binary mixtures to determine the additive or synergistic effects on growth and OTA production by *Aspergillus* section *Nigri* strains under different environmental conditions in peanut kernels. Thus, the aim of the present study was to evaluate the effect of binary mixtures of the antioxidant, butylatedhydroxyanisole, and the antimicrobial, propyl paraben, on (i) the lag phase before growth; (ii) growth rates and (iii) OTA production by strains of *Aspergillus* section *Nigri* under different water activities on peanut meal extract agar.

## 2. Materials and Methods

### 2.1. Fungal strains

Four *Aspergillus* section *Nigri* strains were evaluated: *A. carbonarius* (RCPG and RCP203), *A. niger* aggregate (RCP42 and RCP191). All of the strains were isolated previously from peanut kernels in Argentina [7]. The identification of strains was carried out on the basis of macroscopic and microscopic features of the fungal isolates. Only *A. carbonarius* isolates were identified at species level [30], whilst the other biseriate isolates were, on the whole, classified as *A. niger* aggregate [31]. Ochratoxin A production was assayed on YES medium (2% yeast extract, 15% sucrose) [7]. Strains were maintained in glycerol (15%) at −80 °C and kept in the culture collection at the Department of Microbiology and Immunology, National University of Río Cuarto, Córdoba, Argentina.

### 2.2. Antioxidants

The antioxidant 2(3)-tert-Butyl-4-hydroxyanisole (BHA) and antimicrobial n-propyl 4-hydroxybenzoate (PP) were used and obtained from Sigma-Aldrich Chemical (Dorset, UK). Stock solutions of BHA and PP (1 M) were prepared by dissolving 18 g in 100 mL of ethyl alcohol absolute. 

### 2.3. Culture medium

Peanut meal extract agar (PMEA) was prepared at 3% (w/v).Thirty grams of ground peanuts per liter were boiled for 45 min and filtering the resultant mixture through a double layer of muslin. The volume was made up to 1 L and agar-agar at 2% (w/v) was added [28,29]. The water activity of the basic medium was adjusted to 0.995, 0.980 and 0.930 with known amounts of glycerol [32]. The basic media was autoclaved at 120 °C for 20 min before cooling at 50 °C and poured into 90-mm sterile Petri dishes. From stock solutions of BHA and PP, an appropriate volume was added to autoclaved based media (PMEA) to reach the intended BHA and PP concentrations in each binary mixture: mixture M1 (0.5 + 0.5 mM), M2 (1.0 + 0.5 mM), M3 (2.5 + 0.5 mM), M4 (0.5 + 1.0 mM), M5 (1.0 + 1.0 mM), M6 (2.5 + 1.0 mM), M7 (5.0 + 2.5 mM) and M8 (10 + 2.5 mM). The PMEA medium with the same amount of ethyl alcohol absolute was used as control. Water activity of representative plates of each treatment was checked at the beginning and during the experiment with an AquaLab Series 3 (Decagon Devices, Inc., Pullman, WA, USA). 

### 2.4. Inoculation and incubation conditions

Fungal strains were grown on malt extract agar (MEA) for 7 days at 25 °C to obtain heavily sporulating cultures, and a spot of spores suspended in soft agar was inoculated in the center of each plate. The plates were incubated under all the assayed conditions. Petri dishes of the same a_w_ levels were sealed in polythene bags for maintaining constant a_w_ levels. Four replicate plates per treatment were used and incubated at 25 °C for 4 weeks; all the experiments were repeated twice.

### 2.5. Growth parameters

Assessment of growth was made daily during the incubation period, with peanut meal extract agar cultures being examined using a binocular magnifier (10×). Two diameters of the growing colonies were measured at right angles to each other until the colony reached the edge of the plate. The radii of the colonies were plotted against time, and a linear regression applied to obtain the growth rate (mm/day) as the slope of the line. Lag phase before growth (h) in each treatment was determined as the abscissa from the growth rate curves. 

Number of growth and lag phase analysis = 3, a_w_ × 1, T × 4, strains × 4, rep × 1, antioxidant and antimicrobial mixture × 9, treatments (8 mixtures and 1 control).

### 2.6. Ochratoxin A extraction

At the end of the incubation period (28 days), OTA was determined following the methodology proposed by Bragulat *et al.* [33] with some modifications. From the plates of each treatment, three agar plugs were removed from different points of the colony and extracted with 1 mL of methanol. The mixture was centrifuged at 14,000 rpm for 10 minutes. The solutions were filtered, evaporated to dryness, re-dissolved in 200 µL of mobile phase (acetonitrile-water-acetic acid, 57: 41: 2) and the extract analyzed by high performance liquid chromatography (HPLC). 

### 2.7. Detection and quantification of ochratoxin A

The production of OTA was detected and quantified by the methodology proposed by Scudamore & McDonald, [34] with some modifications, the reversed phase high performance liquid chromatography (HPLC). The HPLC system consisted of a Hewlett Packard model 1100 pump (Palo Alto, CA, USA) connected to a Hewlett Packard 1100 Series with fluorescence detector (λ_exc_ 330 nm; λ_em_ 460 nm) and a data module Hewlett Packard Kayak XA (HP ChemStation Rev. A.06.01). The C_18_ column (Supelcosil LC-ABZ, Supelco; 150 × 4.6 mm, 5 μm particle size), connected to a pre-column (Supelguard LC-ABZ, Supelco; 20 × 4.6 mm, 5 μm particle size) was used. The mobile phase was pumped at 1.0 mL/min. The injection volume was 100 μl and the retention time was around 4 minutes. 

Number of OTA analysis = 3, a_w_ × 1, T × 4, strains × 4, rep × 1, antioxidant and antimicrobial mixture × 9, treatments (8 mixtures and 1 control).

### 2.8. Statistical analysis

Data analyses were done by analysis of variance. All data were transformed to lg (x + 1) to obtain the homogeneity of variance. Means were compared by Fisher´s Least Significant Difference Test to determine the influence of a_w_, antioxidant mixtures concentration and strains on lag phase before growth, growth rate and OTA levels produced by the section *Nigri* strains. The Pearson correlation coefficient was used to evaluate the strength of the relationship between growth rate and OTA levels produced by the strains. The analysis was conducted using PROC GLM in SAS (SAS Institute, Cary, NC) [35]. 

## 3. Results and Discussion

### 3.1. Effect of antioxidants treatments on lag phase and growth rate

The analysis of variance on the effect of single (strains, a_w_ and antioxidant mixtures concentration) two- and three-way interaction showed that all factors alone and all interactions were statistically significant (*P* < 0.0001) in relation to lag phases and growth rates forall *Aspergillus* section *Nigri* strains assayed (Table 1). 

**Table 1:** Analysis of variance of water activity (a_w_), antioxidant mixtures (M) and different isolates (I), and their interactions on lag phase and growth rate of *Aspergillus* section *Nigri* strains at 25 °C.

Source of variation	Df ^a^	Lag phase		Growth rate	
		MS ^b^	F ^c^	MS ^b^	F ^c^
I	3	118472.01	10.24*	47.24	15789.44*
M	7	17221856.84	1493.79*	399.36	99999.99*
a_w_	2	1278196.03	108.61*	40.33	13472.66*
I × M	28	60021.36	5.15*	31.00	10584.69*
I × M × a_w_	78	108999.99	9.33*	10.26	3552.52*

^a^ Degrees of freedom. ^b^ Mean square. ^c^ F-Snedecor. * Significant *P* < 0.0001.

Table 2 shows the effect of mixtures of antioxidants (BHA + PP) in different concentrations on lag phase before growth (h) in four *Aspergillus* section *Nigri* strains. In general, all strains showed a similar behavior. Further, the lag phase increased significantly as a_W_ decreased in control and each antioxidant mixture treatments. The lengthened lag phases were observed in the presence of the highest concentrations (M7 and M8) of both antioxidant and antimicrobial combinations for *A. carbonarius* and *A. niger* aggregate strains. The mixture M2 and M5 showed that an increase in the doses of PP from 0.5 to 1.0 mM resulted in an increase in the lag phase from 31 to 73 h and from 32 to 93 h at 0.980 a_w_ for *A. carbonarius* (RCP 203) and *A. niger* aggregate (RCP 42), respectively (*P* < 0.0001). The mixture M7 BHA + PP (5.0 + 2.5 mM) showed the highest lag phase before growth, 117 and 216 h at 0.995 a_w _for *A. carbonarius* (RCP 203) and *A. niger* aggregate (RCP 191), respectively. At the highest concentration of antioxidants mixture (M8) BHA + PP (10 + 5.0 mM) showed that all the strains tested were not able to reach the exponential phase. 

**Table 2:** Effect of antioxidant mixtures (BHA, butylatedhydroxyanisol; PP, propyl paraben) and water activity (a_w_) on the lag phase of *Aspergillus* section *Nigri* strains on peanut meal extract agar at 25 °C.

Strains	a_w_	Lag phase (h)								
		(BHA + PP) (mM)								
		0	M 1	M 2	M 3	M 4	M 5	M 6	M 7	M 8
RCP G^a^	0.995	11 ^jk^	21 ^lmno^	28 ^fghi^	36 ^h^	28 ^fghi^	41 ^fgh^	68 ^fg^	-	-
	0.980	20 ^klm^	30 ^fghi^	32 ^ghi^	54 ^defghi^	40 ^fgh^	62 ^ij^	79 ^g^	-	-
	0.930	28 ^fghi^	28 ^fghi^	57 ^ij^	118 ^de^	64 ^fg^	67 ^fg^	97 ^fg^	-	-
RCP 203^a^	0.995	15 ^klmno^	27 ^fghi^	22 ^ijklm^	21 l^mno^	45 ^gh^	60 ^ij^	86 ^gh^	117 ^de^	-
	0.980	18 ^lmno^	33 ^fghi^	31 ^fghi^	40 ^fgh^	65 ^fg^	73 ^defg^	86 ^gh^	-	-
	0.930	46 ^gh^	58 ^ij^	49 ^gh^	79 ^defg^	76 ^defg^	80 ^g^	103 ^fg^	-	-
RCP 42^b^	0.995	10 ^jk^	26 ^fghi^	59 ^ij^	53 ^defghi^	77 ^defg^	89 ^gh^	110 ^d^	-	-
	0.980	16 ^klmno^	29 ^fghi^	32 ^fghi^	39 ^fgh^	52 ^defghi^	93 ^fg^	-	-	-
	0.930	27 ^fghi^	51 ^defghi^	59 ^ij^	59 ^ij^	84 ^g^	260 ^a^	-	-	-
RCP 191^b^	0.995	10 ^jk^	13 ^ghi^	18 ^lmno^	26 ^fghi^	26 ^fghi^	40 ^fgh^	45 ^gh^	216 ^b ^	-
	0.980	21 ^lmno^	27 ^fghi^	23 ^ijklm^	29 ^fghi^	38 ^fgh^	34 ^fghi^	60 ^ij^	-	-
	0.930	27 ^fghi^	47 ^gh^	42 ^fgh^	55 ^defghi^	59 ^ij^	95 ^fg^	131 ^c^	-	-

Mean in a row with a letter in common is not significantly different according to LSD test (*P* < 0.0001). (–) Under these conditions the strains were not able to reach the exponential phase.

^a^ *A. carbonarius* (RCP G and RCP 203); ^b^ *Aspergillus niger* aggregate (RCP 42 and RCP 191).

Mixtures (BHA + PP): M1 (0.5 + 0.5 mM), M2 (1.0 + 0.5 mM), M3 (2.5 + 0.5 mM), M4 (0.5 + 1.0 mM), M5 (1.0 + 1.0 mM), M6 (2.5 + 1.0 mM), M7 (5.0 + 2.5 mM) and M8 (10 + 2.5 mM).

**Figure 1:**
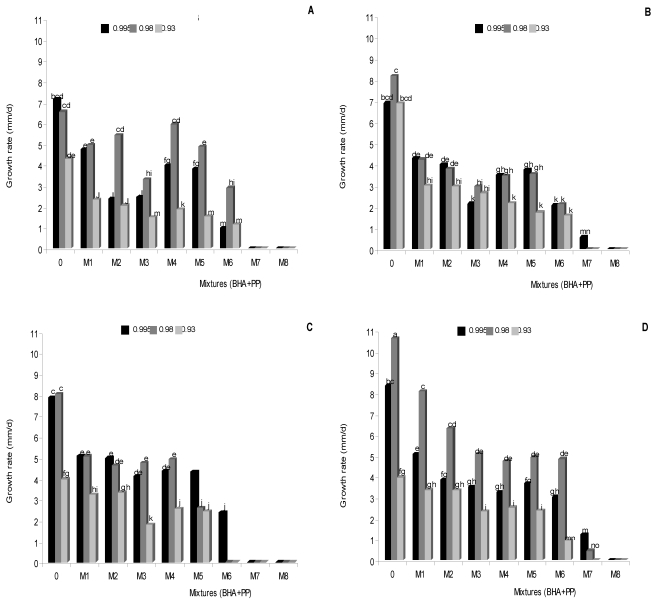
Combined effect of butylatedhydroxyanisol (BHA) and propyl paraben (PP) and water activity (a_w_) on the growth rate of *Aspergillus carbonarius* RCP G (A), RCP 203 (B) and *A. niger* aggregate RCP 42 (C) and RCP 191 (D) on peanut meal extract agar at 25 °C.

Figure 1 shows the effect of antioxidant mixtures (BHA + PP) in different concentrations on the growth rate of *Aspergillus* section *Nigri* strains at different a_W_ levels at 25 °C. The antioxidant mixture M8 (10 + 2.5 mM) completely suppressed mycelial growth for all *Aspergillus* section *Nigri* strains at different a_w_ levels assayed. This behavior was observed with mixture M7 (5.0 + 2.5 mM) on *A. carbonarius* (RCP G) and *A. niger* aggregate (RCP 42) at all a_w_ assayed, and on *A. carbonarius* (RCP 203) at 0.930 and 0.980 and on *A. niger* aggregate (RCP 191) at the lowest a_w_. While with mixture M6 completely suppressed mycelial growth was observed only on *A. niger* aggregate (RCP 42) at 0.930 and 0.980 a_w_. No significant differences between the mixtures BHA + PP M4 (0.5 + 1.0 mM) and M5 (1.0 + 1.0 mM) on growth rate of *A. carbonarius* RCP 203 at all a_w_ assayed and between M3 (2.5 + 0.5 mM) and M4 (0.5 + 1.0 mM) for both *A. niger* aggregate strains at 0.980 and 0.995 a_W_ were observed (*P* < 0.0001). At 0.980 a_w_ the growth rates of *A. carbonarius* RCP G strain were significantly highest in mixtures M2 to M6; whereas in *A. niger* aggregate RCP 191 strain this behavior was observed in control treatment and mixtures M1 to M6 (*P* < 0.0001). 

### 3.2. Effect of antioxidants treatments on ochratoxin A production

**Figure 2:**
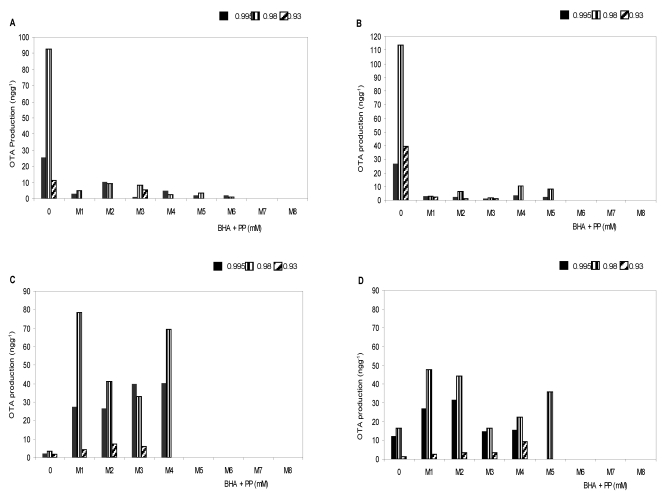
Combined effect of butylatedhydroxyanisol (BHA) and propyl paraben (PP) and water activity (a_w_) on OTA production by *Aspergillus carbonarius* RCP G (A), RCP 203 (B) and *A. niger* aggregate RCP 42 (C), RCP 191 (D) on peanut meal extract agar at 25 °C. Detection limit: 1 ng g^−1^ (ppb). Antioxidant mixtures (BHA + PP): M1 (0.5 + 0.5 mM), M2 (1.0 + 0.5 mM), M3 (2.5 + 0.5 mM), M4 (0.5 + 1.0 mM), M5 (1.0 + 1.0 mM), M6 (2.5 + 1.0 mM), M7 (5.0 + 2.5 mM) and M8 (10 + 2.5 mM).

Recovery of the toxin was 89.2 ± 9.7% from the peanut meal extract agar at tested OTA levels. The detection limit of the technique was 1 ng g^−1^. The effect of the concentration range of BHA + PP mixtures/a_w_ treatment at 25 °C on OTA production for all the tested strains is shown in Figure 2. In general, OTA production did not show a similar pattern to the one on the growth rate. The effect of the antioxidants treatments was variable and depended on the species and the water availability assayed. Ochratoxin A production was significantly reduced in respect of control in both *A. carbonarius* strains with all mixtures. On the other hand, for both *A. niger* aggregate strains, the response varied to the binary mixture assayed. A significant stimulation in OTA production was observed with mixtures M1 to M5 mainly at the highest a_w_, while mixtures M6 (2.5 + 1.0 mM), M7 (5.0 + 2.5 mM) and M8 (10 + 2.5 mM) completely inhibited OTA production in all strains assayed; except to M6 in *A. carbonarius* strain (RCP G). 

The analysis of variance on the effect of single (strains, a_W_ and antioxidant mixture concentration) two- and three- way interaction showed that all factors alone and all interactions were statistically significant (*P* < 0.0001) in relation to OTA production for all *Aspergillus* section *Nigri* strains assayed (Table 3). The Fisher´s Least Significant Difference test (LSD) of data shows the influence of water activity, BHA and PP mixture concentration on growth parameters (lag phase and growth rate) and OTA production. The statistical analysis of all strains showed that all analyzed factors influenced significantly on growth parameters, whereas on OTA production, a_w_ and mixture concentration influenced significantly (*P* < 0.05) (Table 4).

**Table 3:** Analysis of variance of water activity (a_w_), antioxidant mixtures (M) and different isolates (I), and their interactions on OTA production by *Aspergillus* section *Nigri* strains at 25 °C.

Source of variation	OTA production			
	Df ^a^	MS ^b^	F ^c^	Pr > F
I	3	67.65	15159.37*	0.0001
M	7	217.73	48788.22*	0.0001
a_w_	2	29.66	6623.96*	0.0001
I × M	28	9.33	2071.76*	0.0001
I × M × a_w_	78	1.45	322.30*	0.0001

^a^ Degrees of freedom. ^b^ Mean square. ^c^ F-Snedecor. * Significant *P* < 0.0001.

**Table 4:** Influence of water activity (a_w_), and antioxidant mixtures (M)on growth parameters and OTA production at 25 °C.

Treatments	Growth rate (mm d^−1^)	Lag phase (h)	OTA production (ng g^−1^)
	Mean ± SD		
Water activity (a_w_)			
0.995	0.44 ± 0.37 ^a^	1.93 ± 0.68 ^a^	1.59 ± 1.37 ^a^
0.980	0.41 ± 0.33 ^a^	1.97 ± 0.74 ^a^	1.50 ± 1.42 ª
0.930	0.28 ± 0.28 ^b^	2.21 ± 0.76 ^b^	1.09 ± 1.09 ^b^
Antioxidant mixtures BHA + PP (mM)			
M1 (0.5 + 0.5)	5.72 ± 0.26 ^a^	1.16 ± 0.55 ^a^	2.76 ± 2.02 ^a^
M2 (1.0 + 0.5)	3.89 ± 0.16 ^b^	1.55 ± 0.46 ^b^	2.64 ± 1.74 ^a^
M3 (2.5 + 0.5)	1.39 ± 0.21 ^c^	1.88 ± 0.58 ^c^	1.66 ± 1.47 ^b^
M4 (0.5 +1.0)	1.72 ± 0.75 ^c^	1.91 ± 0.99 ^c^	0.45 ± 0.37 ^c^
M5 (1.0 +1.0)	0.87 ± 0.36 ^d^	2.02 ± 1.00 ^c^	0.11 ± 0.07 ^d^
M6 (2.5 +1.0)	0.29 ± 0.16 ^d^	2.36 ± 0.59 ^d^	0.08 ± 0.02 ^d^
M7 (5.0 + 2.5)	0.17 ± 0.14 ^d^	2.22 ± 0.49 ^d^	0.00 ± 0.00 ^d^
M8 (10 + 2.5)	0.00 ± 0.00 ^d^	2.05 ± 1.00 ^e^	0.00 ± 0.00 ^d^

Data were transformed to lg (x + 1). ^a, b, c, d, e^ Groups with different letters are significantly different according to Fisher’s LSD test (*P* < 0.05). SD: standard deviation.

A significant (*p* < 0.05) correlation (r = 0.525, r = 0,715, r = 0,469, r = 0,630 for RCP G, RCP 203, RCP 42 and RCP G, respectively) between the growth rate and OTA production in all *Aspergillus* section *Nigri* strains was found (data not shown).

In this study, it was evaluated whether the different binary mixtures of BHA and PP might be able to inhibit the *Aspergillus* section *Nigri* species growth and OTA production at 25 °C on peanut meal extract agar.

In previous works carried out with these strains and PMEA, it was observed that 20 mM of BHA or 5 mM of PP at 25 °C, completely inhibited both the growth rate and OTA production in all a_W_ condition assayed [28,29]. The results obtained in the present work showed that the combinations of BHA and PP at lower concentrations (M7: 5.0 + 2.5 and M8: 10 + 2.5) than those previously used showed that all the strains tested were not able to reach the exponential phase, completely inhibiting the growth rate of all *Aspergillus* section *Nigri* strains. In addition, the combination of BHA and PP at lower concentrations than each antioxidant separately (M6, M7 and M8) reduced OTA production significantly in all environmental conditions assayed. To discern the effect of antioxidants on lag phase of these fungal species is important because it could prevent the visible fungal growth on grains and consequent mycotoxin production. 

## 4. Discussion

The results obtained regarding OTA production with mixtures M1 to M5 in *A. niger* aggregate strains suggest that the growth control does not necessarily mean that toxin production is also inhibited. In previous works, stimulation production of other mycotoxin in presence of low concentrations of antioxidants has been observed. Fumonisin stimulation has been informed with mixture of BHT + PP (1 mM) by *F. verticillioides* and *F. proliferatum* at 0.995 a_w_ on maize based media [17]. Similar effects have been observed by Nesci *et al.* [36], who observed stimulation of AFB_1_ production by *A. flavus* and *A. parasiticus* strains with some combinations of subinhibitory concentrations of natural phytochemicals (ferulic and cinamic acids) on maize grains at different a_W_ conditions. It has been reported that subinhibitory doses together with inadequate distribution of preservative, especially at low water activities, could enhance fungal growth [37,38]. This behavior was also observed in population of *Aspergillus* species with some concentration of propionate mixtures [39], trihydroxybutyrophenone (THB) [23], peppermint and boldus [40], quercetin and caffeic acid [41]. This behavior suggests that the combinations of antioxidants should be assayed at several concentrations, with various strains and environmental conditions to find safe concentrations and prevent undesirable effects.

Some authors [25], evaluated the effects of phenolic antioxidant in OTA production by *A. carbonarius* on synthetic medium. They showed that some antioxidants, e.g., gallic, 4-hydroxybenzoic and chlorogenic acids tended to inhibit OTA production. In other study with other phenolic compounds, Romero *et al.* [41], observed a significant reduction in growth rate and OTA production with 250 mg L^−1^ of caffeic acid, rutin and quercetin. Recently, these authors [27] reported that the highest concentration of gallic acid (500 mg L^-1^) had significant effect on lag phase and growth rate in *A. carbonarius* strains. Whereas, a significant effect on OTA production was observed even at the lowest concentration (100 mg L^-1^) assayed. 

Many food preservatives and fungicides have been used in combination. Indeed, some works showed that low concentrations of both antioxidants may be more effective together than either one alone. Reynoso *et al.* [17] demonstrated a synergistic effect of the mixture BHA + PP at 0.5 + 1.0 mM to control the growth rate and fumonisin production by *F. verticillioides* and *F. proliferatum* at 0.995 and 0.980 a_W_ on maize based media. In another study, [24] showed that higher concentrations of BHA + PP (20 + 10 or 10 + 20 mM) at 0.982 a_w_ and BHA + PP (10 + 20 or 20 + 10 mM) at 0.955 a_w_ completely inhibited growth rate of *A. flavus* and *A. parasiticus* strains on peanut seeds. In addition, all antioxidant mixtures (BHA + PP, 10 + 10, 10 + 20, 20 + 10 and 20 + 20 mM) significantly reduced AFB_1_ accumulation after 11 and 35 days at 0.982 a_w_ levels for all *Aspergillus* section *Flavi* strains assayed. The results of the present work concur with results of the above mentioned authors who claimed that AFB_1_ production depends on a_w_ levels, incubation time and applied antioxidant mixtures (BHA + PP). In other studies, other antioxidants in combination have been tested as potent fungal inhibitors, and it was observed that benzoic and sorbic acids together are better than alone, inhibiting spoilage yeasts and filamentous fungi [42]. Khan *et al*. [43] showed that the combination of BHA (5 mM) and imazalil (250 mM) reduced the development of anthracnose lesions by 65% after inoculation of banana fruit with *Colletotrichum musae* conidia. 

The peanut grain storage in our country extends from six to eight months and peanuts are subjected to thermal treatment for the production of peanut oil, roasted peanuts and other products, before they reach the consumer. In previous study, Passone *et al*. [44] showed that the levels of BHA and PP in pods had decreased 66 to 75% and 69 to 76% of the initial levels, respectively. This reduction in the antioxidant concentration during the peanuts storage ensures that the residue of antioxidants in this substrate did not exceed the maximum levels established (200 µg g^−1^ fat or oil content of the food product) [45].

Current information about action mechanisms of BHA and PP on fungal species is limited. In general, for phenolic compounds several mechanisms of antifungal activity have been proposed. It has been suggested that BHA affects the cell membrane by changing pH values and affecting transduction energy and substrate transport [46,47]. With respect to parabens, the following antimicrobial mechanisms have been determined: enzymatic function inhibition, lipid membrane dissolution, nutrients transport interference, potential membrane destruction and RNA and DNA synthesis alteration [48]. Recently, Kim *et al.* [49] showed that the antioxidant caffeic acid is a potent antiaflatoxigenic agent in *A. flavus* strains. The action mode of this anti-aflatoxigenic activity appears to be associated with attenuation of the oxidative stress response of the fungus to organic peroxides. However, there is no information about the anti-ochratoxigenic activity of BHA or PP in *Aspergillus* section *Nigri* species. 

## 5. Conclusions

The results obtained in the present study suggest that only determinate combination of BHA and PP antioxidants present additive or synergistic effects on growth rate and OTA production by *Aspergillus* section *Nigri* strains. The binary mixture M7 and M8 (BHA + PP: 5.0 + 2.5 mM and 10 + 2.5 mM) could be the appropriate antioxidants combination to control *Aspergillus* section *Nigri* strains on synthetic media. This behavior could be due to different action mechanisms of these antioxidants on fungal cell at diverse target levels.

These *in vitro* studies must be corroborated by *in situ* conditions on peanut grains before applying the products directly on food commodities. In the future, these antioxidants could be applied in storage peanuts to prolong the hygienic quality of grains and to diminish the entry of ochratoxigenic fungi and OTA into the animal and human food chain.
